# Editorial: Characterizing the neurobehavioral phenotype of mendelian disorders of epigenetic machinery

**DOI:** 10.3389/fgene.2023.1338078

**Published:** 2023-12-05

**Authors:** Rowena Ng, Jacqueline Harris, Tjitske Kleefstra, Angela T. Morgan, Brittany Simpson

**Affiliations:** ^1^ Department of Neuropsychology, Kennedy Krieger Institute, Baltimore, MD, United States; ^2^ Department of Psychiatry and Behavioral Sciences, Johns Hopkins University School of Medicine, Baltimore, MD, United States; ^3^ Kennedy Krieger Institute, Baltimore, MD, United States; ^4^ Department of Genetic Medicine, Pediatrics and Neurology, Johns Hopkins University School of Medicine, Baltimore, MD, United States; ^5^ Department of Human Genetics and Psychiatry, Radboud University Medical Center, Venray, Netherlands; ^6^ Centre of Excellence for Neuropsychiatry, Vincent van Gogh Institute for Psychiatry, Venray, Netherlands; ^7^ Speech and Language, Murdoch Children’s Research Institute, Parkville, VIC, Australia; ^8^ Department of Audiology and Speech Pathology, University of Melbourne, Parkville, VIC, Australia; ^9^ Speech Genomics Clinic, Royal Children’s Hospital, Parkville, VIC, Australia; ^10^ Cincinnati Children’s Hospital Medical Center, Department of Pediatrics, Division of Human Genetics, University of Cincinnati College of Medicine, Cincinnati, OH, United States

**Keywords:** epigenetics, neurodevelopment, cognition, behavior, genetic disorder, neurodevelopmental disorders

Mendelian disorders of the epigenetic machinery (MDEMs) are a group of neurodevelopmental disorders caused by pathogenic variants in genes that encode components of the epigenetic apparatus–writers, erasers or readers of DNA or histone marks and chromatin remodelers (Fahrner and Bjornsson, 2014). These disorders share some common features, including dysregulated growth (71%), and developmental delay and/or intellectual disability (83%) with the majority presenting with both cognitive and growth abnormalities (Fahrner and Bjornsson, 2019). With the increased application of gene sequencing in research and clinical use, the number of identified target genes related to MDEMs have expanded to nearly four times in size since 2015 - with 85 disorders currently known (Harris et al., 2023). Accordingly, there has been a growing recognition that collectively MDEMs may represent a sizeable proportion of individuals with neurodevelopmental disability. Towards this end, research in MDEMs and the associated epigenetic modification–heritable alterations in gene expression without modifications to the DNA sequence–can shed light on the role of epigenetic regulation in the pathogenesis of developmental disorders in addition to typical brain development and growth (Ng et al., 2023).

Systematic research is necessary to better understand the neurobehavioral profile of individual MDEMs, which is an essential step towards disease-specific care management and the development of targeted epigenetic therapies, and to elucidate how epigenetic modification control cognition and behavior in general. To begin moving towards this direction, the goal for this Research Topic was to gather a Research Topic of complementary original research and case series from investigators focused on expanding the cognitive and/or behavioral phenotypes of individual MDEMs.

Kalinousky et al. highlight a study focused on behavioral functioning in individuals with Kabuki syndrome (KS) (MIM #417920), a MDEM caused by a variant in *KMT2D* or *KDM6A* which encodes histone methylation proteins. These authors found a high proportion of child and adult participants with KS met clinical threshold for anxiety based on caregiver-informant inventories, although many already received pharmacologic treatment.

Two publications extend the clinical and cognitive phenotypes of Wiedemann Steiner syndrome (WSS) (MIM #605130), a MDEM caused by a heterozygous variant in *KMT2A*, another histone methyltransferase. Ng et al. report a prospective investigation that employed a battery of parent-rating inventories to index day-to-day behavior functioning in affected individuals. Findings revealed elevated rates of hyperactivity, affective symptoms, behavior regulation challenges, and disruptive sleep behaviors in individuals with WSS. Lin et al. report clinical features of 11 Chinese children with WSS and novel *KMT2A* variants, in comparison to 41 previously reported cases from China and other large cohort studies from Europe and North America. These authors report elevated rate of short stature combined with less aggressive behaviors and feeding difficulties in their sample when compared to WSS cohorts from France and United States. Authors also reported growth trends observed in patients following growth hormone therapy, noting potential therapeutic benefits.

Qu’d et al. report findings from parent questionnaires focused on neuropsychiatric symptoms and adaptive functioning among a large cohort with Rubinstein-Taybi syndrome (RTS) (MIM #180849), a MDEM most commonly caused by a pathogenic variant in *CREBBP* or *EP300,* histone acetyltransferases. In this study, over 80% of their sample reported a clinical level of behavioral problems with more elevated symptoms seen in school-age than later adolescence or adulthood. Interestingly, anxiety disorder was the most common neuropsychiatric diagnosis observed - affecting about a third of the cohort consistently across the life span. The patterns of results from Ng et al., Kalinousky et al., and Qu’d et al. may suggest some overlapping behavioral features (e.g., anxiety) among MDEMs with defects involving the histone machinery which warrant further investigation.

Finally, Nakagawa et al. provides a summary review of the neurobehavioral characteristics seen in mice models of Intellectual Disorder Autosomal Dominant 23 (IDD23) (MIM #615761), with pathogenic variants in *SETD5,* a gene essential in regulating histone acetylation. Cognitive features seen in the mice include atypical fear learning and memory. Aberrant social behaviors such as reduced vocalizations, failure in nest building, and repetitive behaviors were observed regardless of the size of gene deletion, suggesting haploinsufficiency in *SETD5* may contribute to autistic behaviors. Generally, these reported behavioral patterns in mice with *SETD5* variants are in line with trends seen in limited cohort investigations involving human subjects, which suggest elevated behavioral and neuropsychiatric problems inclusive of autism-related behaviors (∼64%) and nearly universal observations of developmental delay (∼93%) (Grozeva et al., 2014; Powis et al., 2018).

In brief, the compilation of publications included in this Research Topic represents the early cornerstone of behavioral phenotyping efforts to understand the role of epigenetic regulation in the origins of neurodevelopmental disorders. Future directions include systematic cross-MDEM investigations, as overlapping neurological patterns may implicate the disease-causing pathways that resulted in commonly affected neural networks (Larizza and Finelli, 2019), and subsequently identification of biomarker targets for epigenetic treatments. Indeed, emergent trends from behavioral investigations suggest select chromatin-related disorders show superimposed cognitive features such as speech/language deficits (St. John et al., 2023), pointing to the molecular effects on neural systems dedicated to language and oromotor functioning. Finally, with more in-depth delineation of syndrome-specific neurobehavioral profiles, investigations focused on correlations between epigenotype and phenotype of individual MDEMs are promising next steps to identify clinical biomarkers of developmental disabilities such as episignatures (i.e., unique DNA methylation patterns) (Sadikovic et al., 2020), which may be utilized to improve early screening, diagnosis and therapeutic response of neurodevelopmental disabilities.
